# Left atrial strain compared to volume in long-term flecainide treated patients with atrial fibrillation – a retrospective cohort study

**DOI:** 10.1186/s12872-026-05886-7

**Published:** 2026-04-21

**Authors:** Alexander Siotis, Pyotr G. Platonov, Bjarne Madsen Hardig

**Affiliations:** 1 Clinical Sciences Helsingborg, Department of Clinical Sciences Lund, Lund University, Svartbrödragränden 3, Helsingborg, 251 87 Sweden; 2 Department of Cardiology, Helsingborg Hospital, Region Skåne, Helsingborg, 252 23 Sweden; 3 Department of Cardiology, Clinical Sciences Lund, Lund University, Lund, 221 85 Sweden; 4 Department of Research and Education, Helsingborg Hospital, Region Skåne, Helsingborg, 252 23 Sweden

**Keywords:** Atrial fibrillation, Flecainide, Rhythm control, Left atrial longitudinal strain, Efficacy, Echocardiography

## Abstract

**Background:**

Left atrial volume index (LAVI) is associated with recurrent atrial fibrillation (AF) during flecainide treatment. Nevertheless, AF patients with normal LAVI experience rhythm control failure. Our study sought to compare the association between left atrial strain and LAVI with rhythm control failure, in flecainide treated patients with AF.

**Methods:**

This retrospective cohort study included consecutive patients ≥ 18 years of age with AF who were discharged after in-hospital initiation of flecainide. Patients without an echocardiogram in sinus rhythm within one year of initiation of flecainide were excluded. The primary endpoint was discontinuations due to rhythm control failure. Receiver operating characteristics determined optimal cutoffs for reservoir (LASr), conduit (LAScd) and contractile (LASct) left atrial longitudinal strains for the primary endpoint. These cutoffs were analysed using Cox regression, with 95% confidence intervals.

**Results:**

Seventy patients were followed for a mean of 1.71 ± 1.56 years (mean age 59.4 ± 11.5 years; 66% male; 8.6% had persistent AF). The area under the curve for LASr was 0.764 (0.595–0.933), for LAScd 0.784 (0.634–0.934), and for LAVI 0.497 (0.345–0.795). Optimal cutoffs for LASr were < 23% and LAScd < 14.5%. These cutoffs had similar specificity (LASr 85% and LAScd 73%) but higher sensitivity (70% and 90%, respectively) compared to ≥moderately increased LAVI (84% and 36%) for the primary endpoint. Hazard ratios for LASr < 23% and LAScd < 14.5% were 9.09 (2.34–35.3) and 18.3 (2.31–145), respectively.

**Conclusions:**

Impaired left atrial strain was associated, independently from LAVI, with discontinuations due to rhythm control failure in patients receiving long-term flecainide treatment for AF.

**Supplementary Information:**

The online version contains supplementary material available at 10.1186/s12872-026-05886-7.

## Introduction

Atrial fibrillation (AF) is a progressive arrhythmia and is a risk factor for heart failure, stroke, morbidity and mortality [1–4]. These risks may be mitigated by an early rhythm control strategy and anticoagulation therapy [5, 6]. Flecainide is a class IC antiarrhythmic drug used for pharmacological cardioversion and long-term rhythm control for patients with AF [7, 8].

Atrial cardiomyopathy is the structural, electrical or functional abnormality of the atria and exhibits a complex, bidirectional relationship with AF and its progression [9]. Structural changes of the atria revealed by an increased left atrial volume index (LAVI) are incrementally associated with AF recurrences and cardioversion failure [10]. Electrical changes in the form of P-wave indices are associated with incident AF as well as with outcomes of long-term flecainide treatment in patients with AF [11, 12].

Left atrial strain echocardiography measures longitudinal changes in muscle fibers, i.e. left atrial function, throughout the atrial cardiac cycle. During ventricular systole the left atrium is filled, stretched and acts as a reservoir (measured as left atrial reservoir strain (LASr)). When the mitral valve opens, the atrium acts like a conduit with passive filling of the left ventricle (conduit strain (LAScd)). Before the valve closes, the left atrium contracts in order to empty the last remaining blood into the left ventricle (contractile strain (LASct)) [13].

Left atrial strain has been shown to predict incident atrial fibrillation, AF disease progression and efficacy outcomes after pulmonary vein isolation (PVI) [14–16]; however, its association with long-term flecainide treatment outcomes remains unknown. In our earlier study, some patients with normal or mildly increased LAVI experienced rhythm control failure during flecainide treatment, while some patients with moderately or severely enlarged atria did not [12]. This may indicate that other underlying factors, such as left atrial function rather than merely left atrial size, may be responsible for rhythm control failure. Therefore, in this sub-study, we hypothesized that left atrial longitudinal strain would show a stronger association with rhythm control failure in patients with AF treated with flecainide. This sub-study sought to: (1) compare the associations between left atrial strain and LAVI and rhythm control failure (as endpoint) in patients with AF receiving flecainide treatment, and (2) explore the relationship between left atrial strain and LAVI.

## Methods

### Study design and participants

**Fig. 1:**
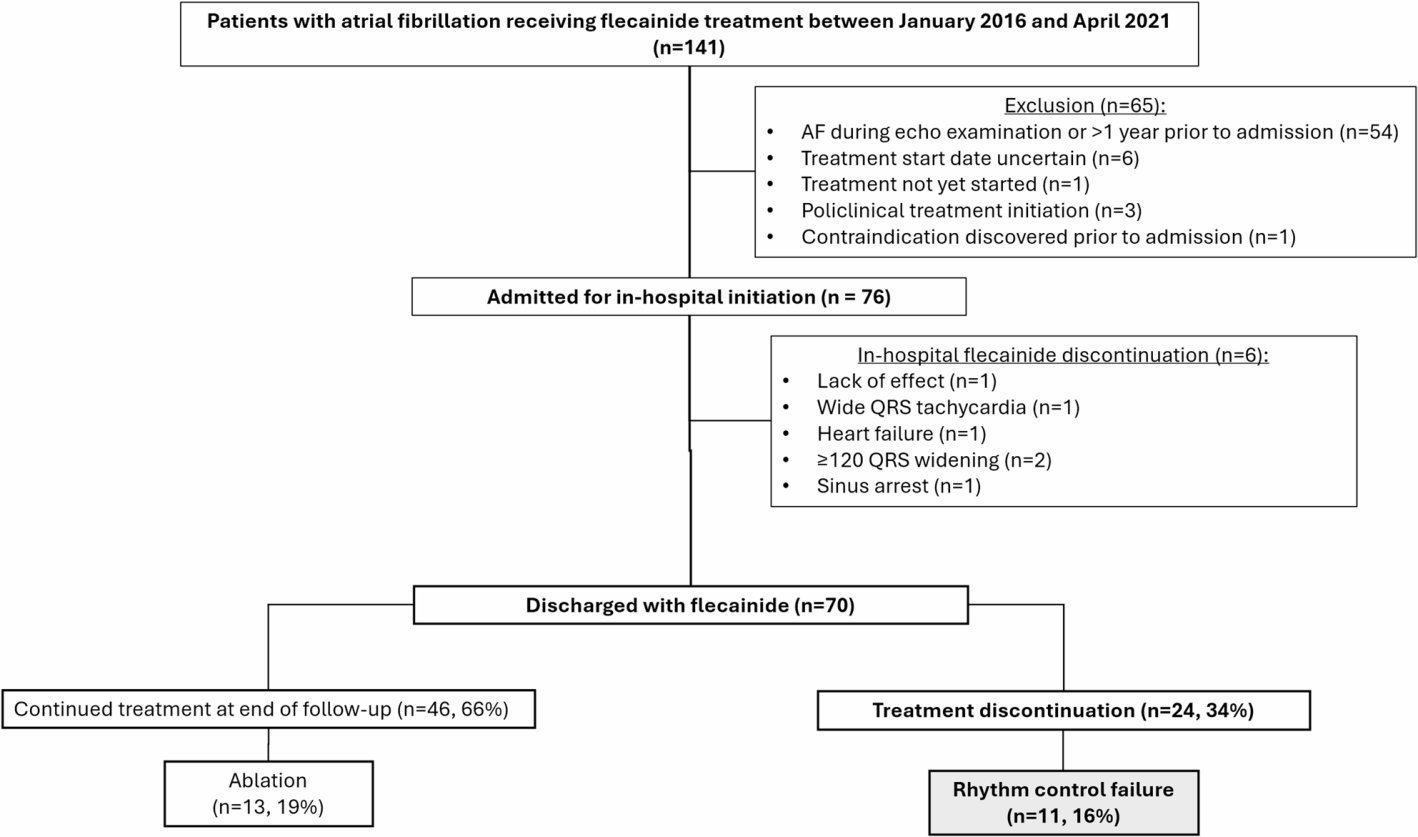
Flowchart of screened, excluded and included patients admitted for in-hospital initiation of flecainide treatment (included patients required sinus rhythm on the patients’ baseline echocardiogram acquired at a maximum 1 year prior to hospital admission and that the patient was discharged with flecainide, *n* = 70), together with distribution of patients who continued treatment and patients who discontinued flecainide treatment due to rhythm control failure (primary endpoint)

This was a sub-study of a previously described retrospective cohort study of patients with AF admitted for in-hospital flecainide initiation as a long-term rhythm control strategy [12]. The inclusion criteria for the main cohort were: consecutive patients of 18 years of age or older with AF who were admitted for in-hospital initiation of flecainide or received at least one dose of flecainide during hospitalization for AF between January 2016 and April 2021, at three hospital sites in southern Sweden. Exclusion criteria for the main cohort were: uncertain treatment initiation date, contraindications discovered before index admission, treatment initiation in the outpatient settings, or follow-up performed outside the three hospitals’ catchment areas. In addition to these exclusion criteria, patients with no echocardiogram in sinus rhythm within one year prior to treatment initiation or patients who were discharged without being prescribed flecainide treatment were excluded (Fig. 1). We did not exclude patients with prior escalated treatment strategies, such as PVI, to allow for a clinically relevant cohort composition as encountered by clinicians in the outpatient clinic. The outpatient and in-hospital screening strategies have been described previously [12]. Patients who were discharged with flecainide had at least yearly clinical follow up visits at the outpatient cardiology clinic that included a 12-lead ECG and relevant blood tests according to the local follow-up protocols. Patients who contacted the outpatient clinic due to symptoms from their AF were scheduled for a subacute extra visit including a 12-lead ECG.

### Data collection

Baseline patient history, characteristics, CHA_2_DS_2_ VASc score prior to flecainide initiation and the following endpoints after hospital discharge were collected retrospectively from the medical records. All patients were started in-hospital on a 100 mg flecainide dose twice daily.

### Echocardiography

Echocardiograms performed within one year before in-hospital initiation of flecainide were collected, and left atrial strain was measured in patients in sinus rhythm (*n* = 70). All echocardiographic measurements were reassessed by the first author (AS), who was blinded to the treatment outcomes, using Philips IntelliSpace Cardiovascular 5 software (LOT 5.2.0.0, 2020-12-01; Philips Medical Systems, Nederland B.V. PC Best, The Netherlands) [12]. The intraclass correlation coefficients (ICCs) for left atrial strain (LASr, LAScd and LASct) of 20 randomly selected cases measured by authors AS and BMH were 0.795 (95% confidence interval [CI] 0.481–0.919), 0.611 (95% CI 0.016–0.846) and 0.386 (95% CI -0.552-0.757), respectively.

The Simpson biplane method of discs was used to calculate left ventricular ejection fraction and left atrial volume. Left atrial volume was corrected for body surface area using Du Bois approximation of body surface area (left atrial volume index, LAVI) [17–19]. LAVI was defined as normal (≤ 34 ml/m^2^), mildly increased (35–41 ml/m^2^) moderately increased (42–48 ml/m^2^), or severely increased (> 48 ml/m^2^) [18].

TOMTEC Imaging Systems GmbH LOT 50.00 2021-11-17 (Unterschleißheim, Germany) was used to perform retrospective, blinded, two-dimensional speckle-tracking strain echocardiography analysis. All left atrial strain analyses were measured in the apical four-chamber view as recommended by the European Association of Cardiovascular Imaging [20]. Manual adjustments of the mitral valve marker were made, but otherwise manual boarder corrections were kept to a minimum, and patients with obvious misaligned borders were deemed to have insufficient data quality for analysis. All strain values are presented as positive values.

### Endpoints

The primary endpoint was flecainide discontinuation due to clinically assessed rhythm control failure (inadequate reduction of symptoms or frequency of AF episodes) by the treating physician in shared decision-making with the patient. Rhythm control failure was detected by either paroxysmal Holter-ECG monitoring, 12 lead ECGs taken at follow up visits or when patients contacted the outpatient clinic because of recurrence of symptoms. The secondary endpoint was flecainide discontinuation due to rhythm control failure due to objectively detected AF on 12 lead ECG or ≥ 30 s of AF on Holter-ECG. No blanking period was applied for the included 70 patients who were discharged after in-hospital screening and flecainide initiation (Fig. 1).

### Statistics

Normally distributed descriptive data are presented as means with standard deviations, and non-normally distributed data are presented as medians with 25–75 interquartile ranges. Two-sided Student’s t-test, Mann-Whitney U-test, Chi square and two-tailed Fishers exact tests were used, as appropriate [21, 22]. Pearson correlation was used to compare the relationship between left atrial strain and LAVI as well as to detect potential collinearity between variables while Kendall’s tau was used for ordinal variables.

Receiver operating characteristic (ROC) curves were used to calculate the areas under the curves (AUC) and to identify the optimal cutoffs for left atrial parameters in relation to the primary endpoint [23]. Univariable Cox regression models were then used for the continuous variables and with the ROC derived cut-offs to measure effect sizes with their 95% confidence intervals for the primary endpoint after hospital discharge [24]. Scatter plots were employed to visualize the relationship between left atrial strain, LAVI, and rhythm control failure (primary endpoint). Sensitivity analyses using Cox regression were performed to assess whether left atrial strain was independent of LAVI, age and sex in relation to the primary endpoint. Patients were censored at the end of follow-up, death, elective PVI, proarrhythmia, side effects, or discovered contraindications.

Reliability analyses were performed with ICCs with a single rating, consistency, 2-way mixed-effects model for the left atrial strain measurements (disclosed above) and has been reported previously for LAVI [12, 25]. All statistical analyses were made in IBM SPSS Statistics for Windows version 29.0.0.0 (241) (Armonk, New York, United States: IBM Corp).

### Ethics

The Swedish Ethical Review Authority approved the study (DNR: 2020–05513), and the study was conducted in accordance with the Declaration of Helsinki.

## Results

### Participants

Seventy patients who were discharged with flecainide were included from the previously-described cohort (Fig. 1) and followed for a mean of 1.71 ± 1.56 years [12]. At baseline, the mean age was 59.4 ± 11.5, two thirds of the patients were male (66%), had previously tried an antiarrhythmic treatment prior to admission (66%) and all patients were symptomatic (Table 1). More than half of the patients had a normal LAVI (58%) and none of the patients had heart failure. Fifty-seven patients were treated with a concomitant betablocker (81%) and one with Verapamil (1.4%) at baseline hospital discharge; four patients ended their betablocker treatment during follow up. At end of follow up eleven patients (16%) had symptoms that triggered discontinuation due to clinically assessed rhythm control failure (primary endpoint). Eight of these eleven patients performed 12-lead ECGs, that verified recurrence of AF. One patient had a Holter ECG revealing ≥ 30 s of AF, one had daily symptoms with concomitant, smart watch detected, high pulse episodes between 150 and 170 beats per minute. One patient experienced worsening symptoms, recognized as AF by the patient, during the first month of flecainide treatment that led to discontinuation. Thus, a total of nine patients (13%) discontinued flecainide treatment due to objectively verified rhythm control failure (secondary endpoint).

**Table 1:** Baseline characteristics of all patients, subdivided into groups containing the patients who experienced adequate rhythm control and rhythm control failure (primary endpoint)

	All *n* = 70	Adequate rhythm control (*n* = 59)	Rhythm control failure (*n* = 11)	*P*-value
Age	59.4 ± 11.5	57.9 ± 11.7	67.4 ± 6.38	0.011*
Male sex, n (%)	46 (66)	43 (73)	3 (27)	0.006*
BMI, kg/m2	26.3 (23.8–30.4)	26.3 (23.6–29.5)	27.2 (24.4–33.5)	0.317
Never smoked, n (%)	39 (60)	35 (65)	4 (36)	0.100
AF phenotype				
* Persistent AF*,* n (%)*	6 (8.6)	5 (8.5)	1 (9.1)	1.00
* Years since AF diagnosis*	2.85 (0.94–6.23)	3.31 (1.04–6.45)	1.76 (0.69–5.87)	0.367
* Days since latest AF*	5 (1–38)	5 (1–43)	2 (0–8)	0.184
* Days since latest spontaneous CV*	42 (3-152)	42 (5-168)	28.5 (2–67)	0.513
* Days since latest CV*	44 (14–238)	68 (15–294)	28 (6–43)	0.101
*Number of prior CVs*,* n (%)*				
* 1–3*	18 (26)	17 (29)	1 (9.1)	0.267
* > 3*	28 (40)	21(36)	7 (64)	0.102
Pulmonary vein isolation, n (%)	5 (7.1)	5 (8.5)	0 (0)	1.00
Hypertension, *n* (%)	23 (33)	17 (29)	6 (55)	0.161
Diabetes, n (%)	1 (1.5)	1 (1.7)	0 (0)	1.00
OAC, n (%)	52 (74)	42 (71)	10 (91)	0.267
CHA2DS2 VASc > 1 point, n (%)	33 (49)	25 (43)	8 (80)	0.042*
EHRA score				
* 1*	0	0	0	N/A
* 2*	41 (59)	34 (58)	7 (64)	1.00
* 3*	24 (34)	22 (37)	2 (18)	0.309
* 4*	5 (7.1)	3 (5.1)	2 (18)	0.173
Prior antiarrhythmic drug use				
* None*,* n (%)*	24 (34)	20 (34)	4 (36)	1.00
* Dronedarone*,* n (%)*	40 (57)	35 (59)	5 (46)	0.511
* Flecainide*,* n (%)*	6 (8.6)	5 (8.5)	1 (9.1)	1.00
* Amiodarone*,* n (%)*	1 (1.4)	1 (1.7)	0 (0)	1.00
* Sotalol*,* n (%)*	2 (2.9)	1 (1.7)	1 (9.1)	0.292
* Disopyramide*,* n (%)*	1 (1.4)	1 (1.7)	0 (0)	1.00
Echocardiography				
* LAVI*,* ml/m*^*2*^	34.1 ± 10.8	33.5 ± 10.3	37.6 ± 13.3	0.253
* LA normal*,* n (%)*	39/67 (58)	33/56 (59)	6/11 (55)	1.00
* LA mildly enlarged*,* n (%)*	15/67 (22)	14/56 (25)	1/11 (9.1)	0.433
* LA moderately enlarged*,* n (%)*	6/67 (9.0)	4/56 (7.1)	2/11 (18)	0.254
* LA severely enlarged*,* n (%)*	7/67 (10)	5/56 (8.9)	2/11 (18)	0.323
* LA reservoir strain*	28.6 ± 10.5	30.6 ± 9.92	20.8 ± 9.44	0.007*
* LASr < 23%*	13/50 (26)	6/40 (15)	7/10 (70)	0.001*
* LA conduit strain*	18.7 ± 8.39	20.3 ± 8.23	12.2 (9.10–14.0)	0.006*
* LAScd < 14.5%*	20/50 (40)	11/40 (28)	9/10 (90)	< 0.001*
* LA contractile strain*	9.91 ± 5.99	10.3 ± 5.76	7.1 (2.63-13.0)	0.291
* LASct < 8.8%*	21/50 (42)	15/44 (38)	6/10 (60)	0.286
* LV end diastolic volume*,* ml*	115 ± 27.0	116 ± 23.7	109 ± 42.1	0.425
* LVEF (biplane)*,* %*	55.1 ± 9.30	54.5 ± 8.96	58.9 ± 10.8	0.170
* E/A-ratio*	1.36 (1.07–1.91)	1.39 (1.05–1.92)	1.34 (1.15–1.91)	0.865
* E/é-ratio*	8.54 ± 2.12	8.56 ± 2.41	8.48 ± 1.28	0.943
* A/á-ratio*	6.81 ± 2.93	6.59 ± 3.00	7.87 ± 2.44	0.266
* TAPSE*	24.3 ± 4.43	24.6 ± 4.72	23.0 ± 2.45	0.324
* Mild mitral regurgitation*,* n (%)*	17 (24)	12 (20)	5 (46)	0.120
* Moderate mitral regurgitation*,* n (%)*	1 (1.4)	1 (1.7)	0	1.00

Patients who reached the primary endpoint were older (mean difference 9.53 years [2.29–16.8], *p* = 0.011), were more likely to be female (73%, *p* = 0.006), and consequently had higher CHA_2_DS_2_ VASc scores (mean difference 1.09 points [0.150–2.03], *p* = 0.024) compared to patients with adequate rhythm control. The baseline echocardiogram was performed at a median of 74 (interquartile range 18–194) days prior to admission. Patients who reached the primary endpoint had significantly lower LASr (mean difference - 9.74% [-16.7-(-2.75)], *p* = 0.007) and LAScd (mean difference - 8.04% [-13.6-(-2.49)], *p* = 0.005) compared to patients with adequate rhythm control. No significant difference could be shown for LAVI (*p* = 0.253) or LASct (*p* = 0.427).

Patients who reached the secondary endpoint were also older (mean difference 11.0 years [3.04-19.0], *p* = 0.007), more likely to be female (67%, *p* = 0.049), had higher CHA_2_DS_2_ VASc scores (mean difference 1.19 [0.139–2.24], *p* = 0.027), had lower LASr (mean difference - 10.3 [-17.9-(-2.68)], *p* = 0.009) and LAScd (mean difference - 7.88 [-14.1-(-1.67)], *p* = 0.014) compared to patients with adequate rhythm control.

Female patients were more likely to be current or previous smokers (65% females vs. 30% males, *p* = 0.006). Five patients had been ablated previously with PVI (three patients [60%] had normal LAVI and the mean age was 66.4 ± 11.7 years), and none of these patients reached the primary nor secondary endpoint. Two of the patients with previous PVI had echocardiograms with insufficient quality to perform strain analysis (40%). Of the remaining three patients one had a LASr < 23% and the other two LASr ≥ 23% (66.7%). A comparison of the baseline characteristics between included and excluded patients is presented in Supplementary Table 1. Excluded patients were more likely to suffer from persistent AF, had significantly higher LAVI, levels of mitral regurgitation but lower LV end diastolic volume compared to included patients. Left atrial strains were also lower, especially in patients where measurements were obtained during AF (LASr obtained in sinus rhythm 28.8 ± 10.7 versus AF 12.7 ± 5.94, p = < 0.001 independent of inclusion into the study cohort).

### Receiver operating characteristics for left atrial strain and left atrial volume index

**Fig. 2:**
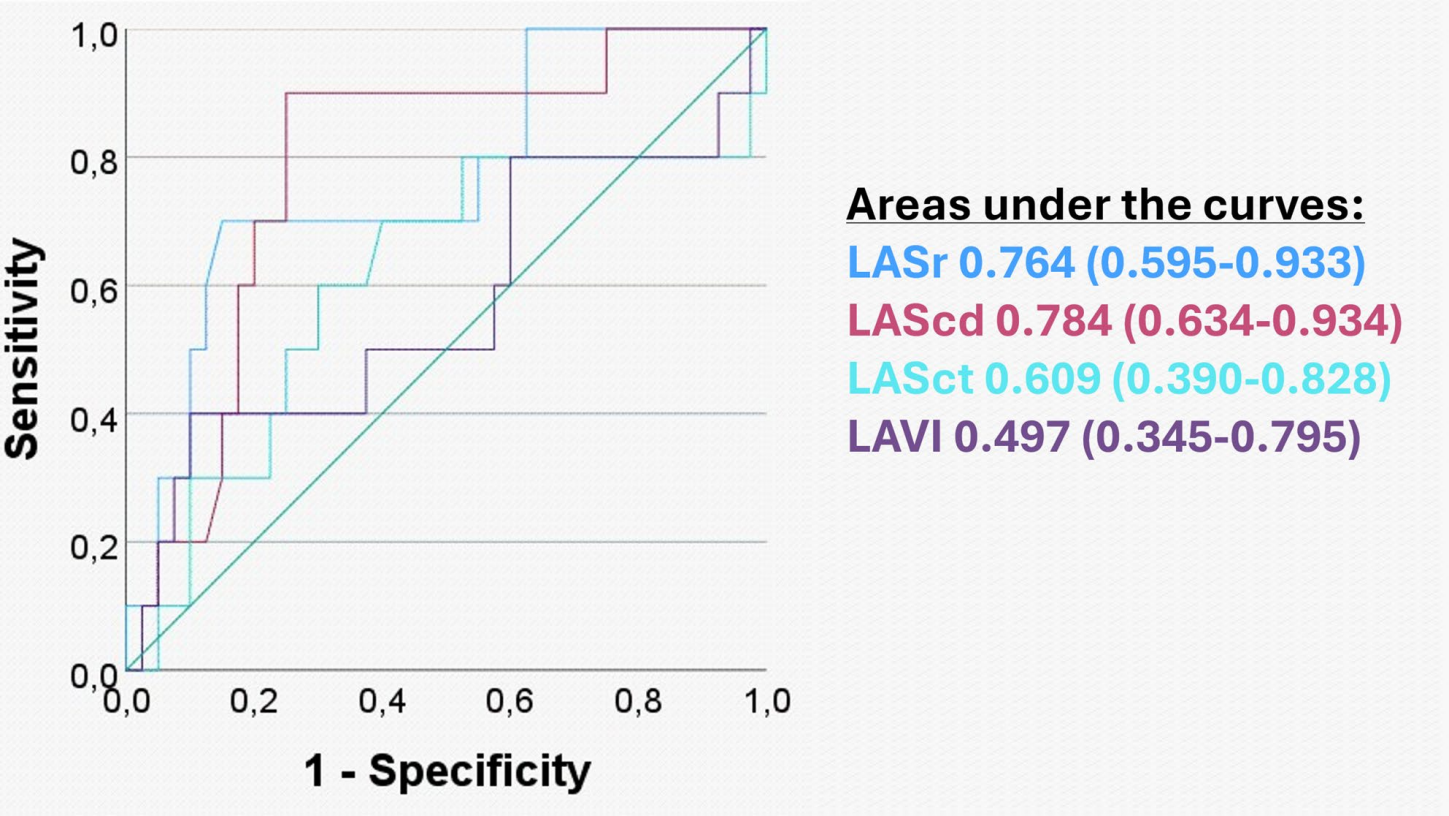
Receiver operating characteristics curves of the left atrial reservoir (LASr), conduit (LAScd), and contractile (LASct) strains as well as of the left atrial volume index (LAVI), with calculations of the respective areas under the curves (AUC) and 95% confidence intervals for discontinuations of flecainide due to rhythm control failure (primary endpoint)

The calculated AUCs for the ROC of the primary endpoint were: LASr 0.764 (95% CI 0.595–0.933), LAScd 0.784 (95% CI 0.634–0.934), LASct 0.609 (95% CI 0.390–0.828) and LAVI 0.497 (95% CI 0.345–0.795). The AUC for the secondary endpoint were: LASr 0.789 (95% CI 0.616–0.962), LAScd 0.772 (95% CI 0.600-0.944), LASct 0.648 (95% CI 0.436–0.861) and LAVI 0.631 (95% CI 0.393–0.870). The optimal exploratory cutoffs for LASr and LAScd were < 23% and < 14.5%, respectively, for the primary endpoint (Fig. 2) as well as for the secondary endpoint (Supplementary Fig. 1).

### Associations between left atrial strain and LAVI with discontinuations due to rhythm control failure

**Fig. 3:**
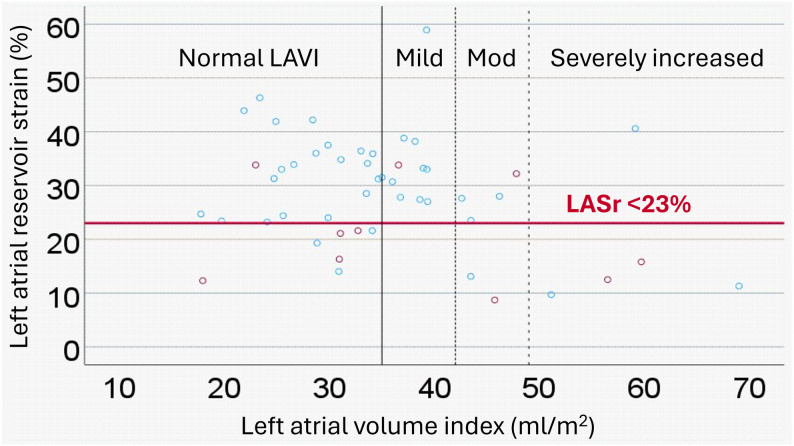
Scatter plot showing the relationship between left atrial reservoir strain (LASr) and left atrial volume index (LAVI). Discontinuations due to rhythm control failure are depicted as red dots. Below the red line are measurements for patients with LASr < 23%, comprising 7 out of the 10 patients (70%) with measurements who discontinued treatment due to rhythm control failure (primary endpoint). Data for patients with normal LAVI (≤ 34 ml/m^2^) are to the left of the bold full line, while data for patients with mildly increased LAVI (35–41 ml/m^2^) are shown immediately to the right of the bold full line. Data for patients with moderately increased LAVI (42–48 ml/m^2^) are shown between the dotted lines, and severely increased LAVI (> 48 ml/m^2^) is shown to the right of the right-most dotted line

**Fig. 4:**
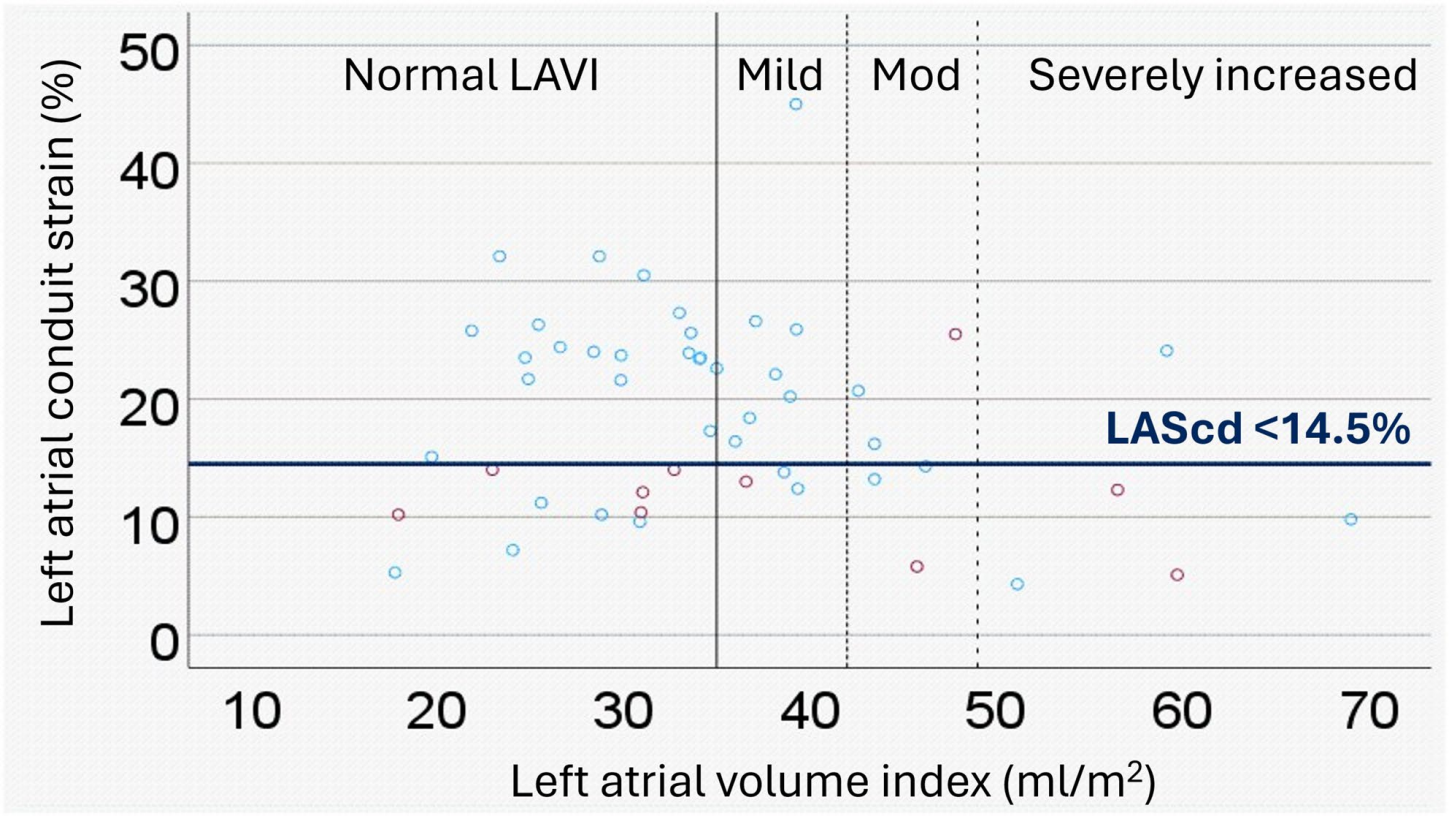
Scatter plot showing the relationship between left atrial conduit strain (LAScd) and left atrial volume index (LAVI). Discontinuations due to rhythm control failure are depicted as red dots. Below the blue line are data for patients with LAScd < 14.5%, comprising 9 out of the 10 patients (90%) with measurements who discontinued their treatment due to rhythm control failure (primary endpoint). Data for patients with normal LAVI (≤ 34 ml/m^2^) are to the left of the bold full line, while data for patients with mildly increased LAVI (35–41 ml/m^2^) are shown immediately to the right of the bold full line. Data for patients with moderately increased LAVI (42–48 ml/m^2^) are shown between the dotted lines, and severely increased LAVI (> 48 ml/m^2^) is shown to the right of the right-most dotted line

LASr as well as LAScd, expressed as positive continuous variables, were inversely associated with both the primary and secondary endpoints, however LASct was not (Table 2 and Supplementary Table 2). Both LASr < 23% and LAScd < 14.5% had higher sensitivity (*n* = 7/10 = 70%, and *n* = 9/10 = 90%) and higher positive and negative predictive values compared to LAVI for the primary endpoint (Table 2; Figs. 3 and 4, respectively). The same pattern was seen for the secondary endpoint as well (Supplementary Table 2). The highest specificity was seen in patients with severely increased LAVI for both primary and secondary end points; however, sensitivity was the lowest in this group. Age, female sex, LASr < 23% and LAScd < 14.5% were significantly associated with the primary and secondary endpoints in the univariable analyses (Table 2 and Supplementary Table 2). Increased LAVI was not associated with the primary endpoint but showed a trend towards the secondary endpoint as a continuous variable and for patients with ≥moderately increased LAVI (Supplementary Table 2). In the sensitivity analyses, LASr and LAScd expressed as continuous, positive variables were independent of LAVI, age and sex in relation to the primary endpoint and did not display more than weak correlation (r^2^) with any of the variables above (Supplementary Table 3). The correlation between LASr and LAScd with baseline CHA_2_DS_2_ VASc scores were also weak (Kendall’s tau for LASr= -0.327, *p* = 0.003 and for LAScd= -0.339, *p* = 0.002, respectively).

**Table 2:** Test accuracy and hazard ratios (HR) for left atrial strain, left atrial volume index (LAVI), age and female sex for the primary endpoint

	Sensitivity	Specificity	PPV	NPV	Accuracy	HR (univariable)	*p*-value
LASr	*N*/A	*N*/A	*N*/A	*N*/A	*N*/A	0.913 (0.853–0.976)	0.008*
LAScd	N/A	N/A	N/A	N/A	N/A	0.889 (0.813–0.972)	0.010*
LASct	N/A	N/A	N/A	N/A	N/A	0.954 (0.858–1.06)	0.389
LASr < 23%	70%	85%	54%	92%	82%	9.09 (2.34–35.3)	0.001*
LAScd < 14.5%	90%	73%	45%	97%	76%	18.3 (2.31–145)	0.006*
LASct < 8.8%	60%	63%	29%	86%	62%	2.20 (0.621–7.81)	0.222
LAVI	N/A	N/A	N/A	N/A	N/A	1.04 (0.984–1.09)	0.170
*Normal LAVI*	55%	41%	15%	82%	43%	0.808 (0.247–2.65)	0.725
≥*Mildly increased LAVI*	46%	59%	18%	85%	57%	1.24 (0.378–4.06)	0.725
≥*Moderately increased LAVI*	36%	84%	31%	87%	76%	2.61 (0.761–8.92)	0.127
*Severely increased LAVI*	18%	91%	29%	85%	79%	2.32 (0.500–10.8)	0.282
Age, years	N/A	N/A	N/A	N/A	N/A	1.09 (1.02–1.17)	0.015*
Female sex	N/A	N/A	N/A	N/A	N/A	6.27 (1.66–23.7)	0.007*

*HR* Hazard ratio with 95% confidence intervals and *p*-values calculated from univariable Cox regression, *LASr* Left atrial reservoir strain, *LAScd* left atrial conduit strain, *LASct* left atrial contractile strain, *LAVI* left atrial volume index (35-41 ml/m^2^ mildly, 42–48 ml/m^2^ moderately and > 48 ml/m^2^ severely increased), *N/A* Not applicable, *NPV* Negative predictive value, *PPV* Positive predictive value

Figures 3 and 4 outline the associations between LAVI, LASr < 23% and LAScd < 14.5%. Of the 11 patients who reached the primary endpoint, five had a normal LAVI (*n* = 3/5 had LASr < 23%, *n* = 5/5 had LAScd < 14.5%), one had a mildly increased LAVI (*n* = 0/1 had LASr < 23%, *n* = 1/1 had LAScd < 14.5%), two had a moderately increased LAVI (*n* = 1/2 had a LASr < 23%, *n* = 1/2 had a LAScd < 14.5%), and two had a severely increased LAVI (*n* = 2/2 had a LASr < 23%, *n* = 2/2 had a LAScd < 14.5%), and one patient did not have an echocardiography of sufficient quality for measurements.

### Relationship between left atrial strain and LAVI

Patients with increased LAVI did not show any significant difference in their LASr (mean difference 1.87 [-4.15-7.90], *p* = 0.535), LAScd (mean difference − 1.97 [-6.77-2.83], *p* = 0.413), LASct (mean difference 0.115 [-3.34-3.56], *p* = 0.947) compared to patients with normal LAVI. LASr and LAVI showed a significant, weak negative correlation (*r*= -0.283 [-0.520-(-0.005), *p* = 0.046], as illustrated in Fig. 3. The correlation between LAScd and LAVI was not significant (Pearson r: 0.170 [-0.114-0.428, *p* = 238]), as illustrated in Fig. 4.

## Discussion

This study revealed that impaired left atrial strain, specifically LASr and LAScd measured in sinus rhythm, was associated with discontinuations due to rhythm control failure in patients with non-valvular, predominantly paroxysmal AF, without significant structural heart disease or substantial left atrial enlargement, receiving long-term flecainide treatment. Exploratory ROC-derived cutoffs revealed that LASr < 23% and LAScd < 14.5% were associated with discontinuations due to rhythm control failure (for both primary and secondary endpoints). These associations were independent of LAVI, age and sex according to the sensitivity analysis. LASct did not reveal any association with flecainide discontinuations due to rhythm control failure and exhibited a low inter-operator reliability. Due to the exploratory nature of these cutoffs above, external prospective validation is needed before clinical decision making and implementation in clinical practice can be made.

Although exploratory in this study, left atrial reservoir strain < 23% has previously been shown in a large Danish cohort study with healthy participants to predict incident AF [26]. In another study of young patients receiving long-term flecainide treatment post mitral valvular replacement due to rheumatic mitral stenosis, LASr > 21% predicted successful rhythm control at 6 months follow-up [27]. In studies of patients with AF and low CHA_2_DS_2_ VASC scores undergoing ablation with PVI, LASr values < 18.8% were shown to predict higher rates of AF recurrence [28].

We have previously shown that both ECG derived P-wave and echocardiographic indices of atrial cardiomyopathy, including LAVI, were associated with flecainide discontinuations due to rhythm control failure in patients receiving long-term flecainide treatment [12]. Our current study revealed that left atrial strain as a measure of left atrial function was associated with flecainide discontinuations due to rhythm control failure. Indeed, patients with normal LAVI but LASr < 23% or LAScd < 14.5% were associated with discontinuations due to rhythm control failure to a higher extent, as shown in Figs. 3 and 4 as well as Supplementary Figs. 2 and 3. As stated above, these exploratory cutoffs need to be validated prospectively before clinical implementation.

LASr and LAScd revealed to have higher AUC values (LASr 0.764 [0.595–0.933] and LAScd 0.784 [0.634–0.934]), high similar specificity (LASr 85% and LAScd 73%) and higher sensitivity (70% and 90% respectively) as compared to LAVI (AUC 0.497 [0.345–0.795] for the primary end point, Table 2; Fig. 2). The AUC values for the secondary endpoint were similar to the values for the primary endpoint (Supplementary Fig. 1). The negative predictive values for LASr < 23% (92%) and LAScd < 14.5% (97%) were higher than those for LAVI (normal LAVI 82%, ≥mildly increased 85%, ≥moderately increased 87% and severely increased 85%) for the primary end point. However, the positive predictive values for LASr < 23% and LAScd < 14.5% remained low to moderate (54% and 45% for the primary endpoint, respectively), likely due to the relatively low absolute number of rhythm control failures in this cohort. Indeed, for patients with normal LAVI, a value of LASr < 23% and LAScd < 14.5% revealed to be both sensitive and highly specific for discontinuation due to rhythm control failure (primary endpoint). Also, on a group level, patients with severely increased LAVI had lower LASr and LAScd values, suggesting that atrial size may be a late sign of atrial dysfunction in atrial cardiomyopathy. These observations are in line with the levels of atrial cardiomyopathy outlined in the recently published international clinical consensus statement on atrial cardiomyopathy [9] and emerging evidence [29]. Indeed, our study suggests that impaired left atrial strain (equivalent to at least moderate atrial cardiomyopathy) seems to be an earlier sign of an unfavourable atrial substrate for flecainide treatment than severely increased LAVI (equivalent to severe atrial cardiomyopathy).

Patients with increased LAVI were incrementally more likely to exhibit lower LASr values (Fig. 3), although the correlation was weak. For LAScd, no significant correlation was revealed (Fig. 4). Previous studies have shown that lower left atrial strain values could be influenced by left ventricular filling pressures, and that left atrial strain strongly correlates with left atrial fibrosis [30–32]. In contrast, prior studies report a low correlation between left atrial volume and degree of left atrial fibrosis [33, 34]. Left atrial fibrosis is the hallmark of atrial cardiomyopathy, which decreases left atrial function [32], and atrial fibrosis is an important substrate for maintaining atrial fibrillation [35]. In this study, decreased left atrial function (i.e. impaired left atrial strain obtained in sinus rhythm) was associated with discontinuations due to rhythm control failure in patients with predominantly paroxysmal AF receiving flecainide treatment.

Age and female sex were, in this study, associated with discontinuations due to rhythm control failure. Our previous study from the original cohort of 130 patients, showed that the association between female sex and flecainide treatment discontinuation was confounded by older age [12]. Patients with higher CHA_2_DS_2_ VASc scores were significantly more likely to reach both the primary and secondary endpoints. While female sex and older age are the most likely reasons for this association, it is possible that this is due to a greater burden of comorbidities as well.

### Strengths and limitations

One of the strengths of this study was that all echocardiograms were reassessed by one of the authors. In addition, the echocardiograms exhibited moderate to good ICC reliability for two of the three left atrial strain measurements (LASr and LAScd) and, as previously reported, excellent reliability for LAVI [12]. The low LASct reliability (ICC 0.386) may have influenced the results and consequently the conclusions from this study on the utility of strain imaging are mainly drawn from the LASr (ICC 0.795) and LAScd (ICC 0.611) measurements. Prior studies have shown LASr to be the most reproducible of the left atrial strain measurements [36, 37]; however, the absolute ICC values reported for LAScd and LASct were significantly higher [37]. The reason for the lower values is likely due to selection of different study images of the left atrium (although from the same examination time point) and definition of the timing of the left atrial contraction phase.

The retrospective nature of this study limited the ability to establish causation. Furthermore, the patient cohort predominantly included patients treated with flecainide against paroxysmal AF, without significant structural heart disease or substantial left atrial enlargement, and the study conclusions are thus mainly applicable to patients with these characteristics. The relatively small sample size with few endpoint events is also a limitation, especially for the statistical robustness of the ROC-based exploratory cutoffs. The exploratory ROC-based cutoffs used are at risk of overfitting the model to the specific data set. These exploratory cutoffs warrant validation in prospective cohorts to strengthen the specific cutoffs for prediction of treatment outcomes in long-termed flecainide treated patients with AF before implementation into clinical practice.

Furthermore, the primary endpoint - relying on discontinuation based on clinically assessed rhythm control failure in shared decision-making with the patient – is subjective and may be prone to detection bias. However, this primary endpoint reflects real world outcomes in a population of patients with symptomatic AF and their clinical response to flecainide treatment. To strengthen the findings and conclusions a secondary endpoint relying on objectively verified AF was added with similar findings as compared to the primary endpoint.

## Conclusions

Impaired LASr and LAScd obtained in sinus rhythm were associated with flecainide discontinuations due to rhythm control failure in patients with non-valvular, predominantly paroxysmal atrial fibrillation without significant structural heart disease. These associations were independent of left atrial volume index, and the correlation between left atrial strain and left atrial volume index was weak. Further prospective studies are needed to validate the specific, exploratory cutoffs generated for left atrial strain in this setting.

## Electronic Supplementary Material

Supplementary Material 1. Supplementary Table 1: Comparison of baseline characteristics between included and excluded patients. Included patients were all in sinus rhythm during their baseline echocardiogram performed at a maximum of 1 year prior to index admission. Thirty-three patients were excluded due to atrial fibrillation during the time of the echocardiogram, and a total of 24 patients had missing left atrial strain values due to inadequate echocardiographic quality or incompatibility with the software used to acquire strain measurements. The results from the total cohort (included and excluded patients) have been published elsewhere previously [12]. Supplementary Table 2: Test accuracy and hazard ratios (HR) for left atrial strain, left atrial volume index (LAVI), age and female sex for the secondary endpoint. Supplementary Table 3: Sensitivity analyses, using Cox regression to calculate the hazard ratios of left atrial reservoir strain (LASr) and left atrial conduit strain (LAScd) for the primary outcome (flecainide discontinuation due to rhythm control failure). LASr and LAScd expressed as positive, continuous variables were independent of left atrial volume index (LAVI), age and sex in relation to rhythm control failure (primary outcome). The hazard ratios for LASr and LAScd with their 95% confidence intervals and *p*-values are displayed in the table. At most, weak collinearity was detected after analysis with Pearsons r^2^ for the variables presented in the table. Supplementary Figure 1: Receiver operating characteristics curves of left atrial strain during the cardiac cycle and left atrial volume index (LAVI) with calculations of the respective areas under the curves (AUC) and 95% confidence intervals for discontinuation of flecainide due to ECG or Holter verified rhythm control failure (secondary endpoint). Supplementary Figure 2: Scatter plot showing the relationship between left atrial reservoir strain (LASr) and left atrial volume index (LAVI). Discontinuations due to ECG or Holter ECG verified rhythm control failures (secondary endpoint) are depicted as red dots. Below the horizontal line are measurements for patients with LASr <23%, comprising 6 out of the 8 patients (75%) with measurements who discontinued treatment due to ECG or Holter ECG verified rhythm control failures (secondary endpoint). Supplementary Figure 3: Scatter plot showing the relationship between left atrial conduit strain (LAScd) and left atrial volume index (LAVI Discontinuations due to ECG or Holter ECG verified rhythm control failures (secondary endpoint) are depicted as red dots. Below the blue line are data for patients with LAScd <14.5%, comprising 7 out of the 8 patients (88%) with measurements who discontinued their treatment due to ECG or Holter ECG verified rhythm control failures (secondary endpoint).

## Data Availability

The datasets generated and/or analysed during the current study are not publicly available due to medical confidentiality and the sensitivity of the data obtained from the patients in the cohort but are available from the corresponding author on reasonable request.

## Abbreviations

AF: Atrial fibrillation
AUC: Area under the curve
CI: Confidence interval
CV: Cardioversion
ECG: Electrocardiogram
EHRA score: European Heart Rhythm Association score
ICC: Intraclass correlations coefficient
LAVI: Left atrial volume index
LASr: Left atrial reservoir strain
LAScd: Left atrial conduit strain
LASct: Left atrial contractile strain
LV: Left ventricular
LVEF: Left ventricular ejection fraction
NPV: Negative predictive value
OAC: Oral anticoagulation
PPV: Positive predictive value
PVI: Pulmonary vein isolation
ROC: Receiver operating characteristics
TAPSE: Tricuspid annular plane systolic excursion
