# Supplementary material for: Left atrial strain compared to volume in long-term flecainide treated patients with atrial fibrillation – a retrospective cohort study
Source: BMC Cardiovasc Disord. 2026 Apr 21;26:344. doi: 10.1186/s12872-026-05886-7 (PMC13101192; doi:10.1186/s12872-026-05886-7)
Supplement: Supplementary file 1 — Supplementary Material 1. Supplementary Table 1: Comparison of baseline characteristics between included and excluded patients. Included patients were all in sinus rhythm during their baseline echocardiogram performed at a maximum of 1 year prior to index admission. Thirty-three patients were excluded due to atrial fibrillation during the time of the echocardiogram, and a total of 24 patients had missing left atrial strain values due to inadequate echocardiographic quality or incompatibility with the software used to acquire strain measurements. The results from the total cohort (included and excluded patients) have been published elsewhere previously [12]. Supplementary Table 2: Test accuracy and hazard ratios (HR) for left atrial strain, left atrial volume index (LAVI), age and female sex for the secondary endpoint. Supplementary Table 3: Sensitivity analyses, using Cox regression to calculate the hazard ratios of left atrial reservoir strain (LASr) and left atrial conduit strain (LAScd) for the primary outcome (flecainide discontinuation due to rhythm control failure). LASr and LAScd expressed as positive, continuous variables were independent of left atrial volume index (LAVI), age and sex in relation to rhythm control failure (primary outcome). The hazard ratios for LASr and LAScd with their 95% confidence intervals and p-values are displayed in the table. At most, weak collinearity was detected after analysis with Pearsons r2 for the variables presented in the table. Supplementary Figure 1: Receiver operating characteristics curves of left atrial strain during the cardiac cycle and left atrial volume index (LAVI) with calculations of the respective areas under the curves (AUC) and 95% confidence intervals for discontinuation of flecainide due to ECG or Holter verified rhythm control failure (secondary endpoint). Supplementary Figure 2: Scatter plot showing the relationship between left atrial reservoir strain (LASr) and left atrial volume index (LAVI). Discont [file 12872_2026_5886_MOESM1_ESM.docx]

**Supplementary materials**

**Supplementary Table 1**

**Supplementary Table 1: Comparison of baseline characteristics between included and excluded patients. Included patients were all in sinus rhythm during their baseline echocardiogram performed at a maximum of 1 year prior to index admission. Thirty-three patients were excluded due to atrial fibrillation during the time of the echocardiogram, and a total of 24 patients had missing left atrial strain values due to inadequate echocardiographic quality or incompatibility with the software used to acquire strain measurements. The results from the total cohort (included and excluded patients) have been published elsewhere previously (12).**

|  | **Included patients (n=70)** | **Excluded patients (n=60)** | **P-value** |
| --- | --- | --- | --- |
| Age | 59.4 ±11.5 | 61.3±12.1 | 0.342 |
| Male sex, n (%) | 46 (66) | 38 (63) | 0.777 |
| BMI, kg/m2 | 26.3 (23.8-30.4) | 27.8 (24.4-32.5) | 0.166 |
| Never smoked, n (%) | 39 (60) | 26 (46) | 0.112 |
| **AF phenotype** |  |  |  |
| *Persistent AF, n (%)* | 6 (8.6) | 31 (51.7) | <0.001* |
| *Years since AF diagnosis* | 2.85 (0.94-6.23) | 2.32 (1.09-6.77) | 0.809 |
| *Number of prior CVs, n (%)* |  |  |  |
| *1-3* | 18 (26) | 20 (35) | 0.280 |
| *>3* | 28 (40) | 20 (35) | 0.521 |
| Pulmonary vein isolation, n (%) | 5 (7.1) | 5 (8.3) | 0.800 |
| Hypertension, n (%) | 23 (33) | 23 (39) | 0.507 |
| Diabetes, n (%) | 1 (1.5) | 4 (6.7) | 0.185 |
| CHA2DS2 VASc >1 point, n (%) | 33 (49) | 28 (48) | 0.904 |
| **Prior antiarrhythmic drug use** |  |  |  |
| *None, n (%)* | 24 (34) | 17 (28) | 0.467 |
| **Echocardiography** |  |  |  |
| *LAVI, ml/m^2^* | 34.1±10.8 | 39.9±12.2 | 0.010* |
| *LA normal, n (%)* | 39/67 (58) | 16/43 (37) | 0.032* |
| *LA mildly enlarged, n (%)* | 15/67 (22) | 13/43 (30) | 0.357 |
| *LA moderately enlarged, n (%)* | 6/67 (9) | 7/43 (16) | 0.246 |
| *LA severely enlarged, n (%)* | 7/67 (10) | 7/43 (16) | 0.371 |
| *LA reservoir strain* | 28.6±10.5 | 17.1±10.7 | <0.001* |
| *LASr <23%* | 13/50 (26) | 27/34 (79) | <0.001* |
| *LA conduit strain* | 18.7±8.39 | 15.2±7.02 | 0.050 |
| *LAScd <14.5%* | 20/50 (40) | 19/34 (56) | 0.152 |
| *LA contractile strain* | 9.91±5.99 | 2.29±6.67 | <0.001* |
| *LASct <8.8%* | 21/50 (42) | 29/34 (85) | <0.001* |
| *LV end diastolic volume, ml* | 115±27.0 | 101±24.3 | 0.005* |
| *LVEF (biplane), %* | 55.1±9.30 | 53.0±8.21 | 0.211 |
| *Mild mitral regurgitation, n (%)* | 17/70 (24) | 14/46 (30) | 0,.464 |
| *Moderate mitral regurgitation, n (%)* | 1/70 (1.4) | 5/46 (10) | 0.035* |

**Supplementary Table 2**

**Supplementary Table 2**: Test accuracy and hazard ratios (HR) for left atrial strain, left atrial volume index (LAVI), age and female sex for the secondary endpoint.

|  | **Sensitivity** | **Specificity** | **PPV** | **NPV** | **HR (univariable)** | **p-value** |
| --- | --- | --- | --- | --- | --- | --- |
| LASr | N/A | N/A | N/A | N/A | 0.904 (0.837-0.976) | 0.010* |
| LAScd | N/A | N/A | N/A | N/A | 0.891 (0.808-0.983) | 0.021* |
| LASct | N/A | N/A | N/A | N/A | 0.933 (0.828-1.05) | 0.249 |
| LASr <23% | 75% | 85% | 50% | 94% | 12.3 (2.48-61.2) | 0.002* |
| LAScd <14.5% | 88% | 73% | 39% | 97% | 15.0 (1.84-122) | 0.011* |
| LASct <8.8% | 38% | 38% | 11% | 75% | 0.398 (0.095-1.67) | 0.207 |
| LAVI | N/A | N/A | N/A | N/A | 1.06 (0.999-1.11) | 0.055 |
| *Normal LAVI* | 56% | 41% | 13% | 85% | 0.842 (0.226-3.14) | 0.797 |
| ≥*Mildly increased LAVI* | 44% | 59% | 15% | 87% | 1.19 (0.319-4.43) | 0.797 |
| ≥*Moderately increased LAVI* | 44% | 84% | 31% | 90% | 3.69 (0.987-13.8) | 0.052 |
| *Severely increased LAVI* | 22% | 91% | 29% | 88% | 3.03 (0.628-14.7) | 0.167 |
| Age, years | N/A | N/A | N/A | N/A | 1.12 (1.03-1.21) | 0.011* |
| Female sex | N/A | N/A | N/A | N/A | 4.86 (1.22-19.5) | 0.025* |
| HR=Hazard ratio with 95% confidence intervals and p-values calculated from univariable Cox regression, LASr=Left atrial reservoir strain, LAScd=left atrial conduit strain, LASct=left atrial contractile strain, LAVI=left atrial volume index (>34 ml/m^2^, 42-48 ml/m^2^ moderately and >48 ml/m^2^ severely increased), N/A=Not applicable, NPV=Negative predictive value, PPV=Positive predictive value. | | | | | | |

**Supplementary Table 3**

Supplementary Table 3: Sensitivity analyses, using Cox regression to calculate the hazard ratios of left atrial reservoir strain (LASr) and left atrial conduit strain (LAScd) for the primary outcome (flecainide discontinuation due to rhythm control failure). LASr and LAScd expressed as positive, continuous variables were independent of left atrial volume index (LAVI), age and sex in relation to rhythm control failure (primary outcome). The hazard ratios for LASr and LAScd with their 95% confidence intervals and p-values are displayed in the table. At most, weak collinearity was detected after analysis with Pearsons r^2^ for the variables presented in the table.

|  | **Age** | **P-value** | **Sex (female)** | **P-value** | **LAVI** | **P-value** |
| --- | --- | --- | --- | --- | --- | --- |
| **Cox regression** |  |  |  |  |  |  |
| LASr | 0.927 (0.863-0.995) | 0.035 | 0.921 (0.862-0.984) | 0.014 | 0.914 (0.847-0.985) | 0.019 |
| LAScd | 0.907 (0.824-0.999) | 0.047 | 0.907 (0.832-0.989) | 0.026 | 0.893 (0.815-0.979) | 0.016 |
| **Pearsons r^2^** |  |  |  |  |  |  |
| LASr | 0.215 | <0.001 | 0.049 | 0.124 | 0.080 | 0.046 |
| LAScd | 0.297 | <0.001 | 0.071 | 0.061 | 0.029 | 0.238 |

**Supplementary Figure 1**

**Supplementary Figure 1**: Receiver operating characteristics curves of left atrial strain during the cardiac cycle and left atrial volume index (LAVI) with calculations of the respective areas under the curves (AUC) and 95% confidence intervals for discontinuation of flecainide due to ECG or Holter verified rhythm control failure (secondary endpoint).


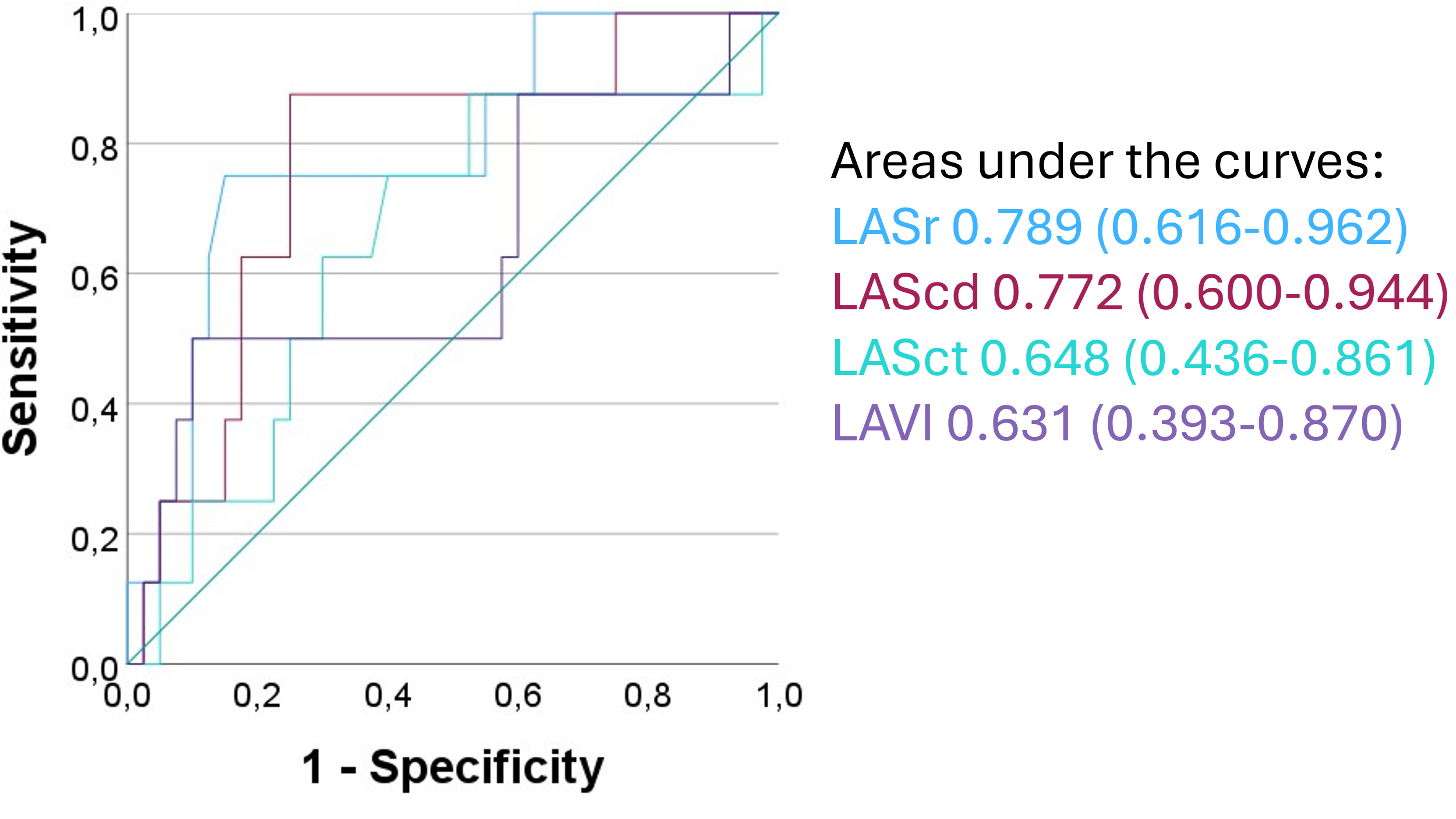


**Supplementary Figure 2**

Supplementary Figure 2: Scatter plot showing the relationship between left atrial reservoir strain (LASr) and left atrial volume index (LAVI). Discontinuations due to ECG or Holter ECG verified rhythm control failures (secondary endpoint) are depicted as red dots. Below the horizontal line are measurements for patients with LASr <23%, comprising 6 out of the 8 patients (75%) with measurements who discontinued treatment due to ECG or Holter ECG verified rhythm control failures (secondary endpoint).

**
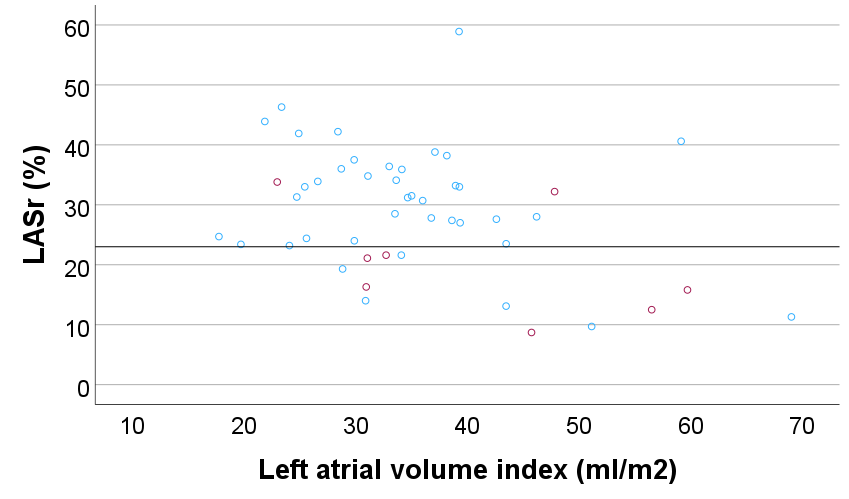
**

**Supplementary Figure 3**

Supplementary Figure 3: Scatter plot showing the relationship between left atrial conduit strain (LAScd) and left atrial volume index (LAVI Discontinuations due to ECG or Holter ECG verified rhythm control failures (secondary endpoint) are depicted as red dots. Below the blue line are data for patients with LAScd <14.5%, comprising 7 out of the 8 patients (88%) with measurements who discontinued their treatment due to ECG or Holter ECG verified rhythm control failures (secondary endpoint).**
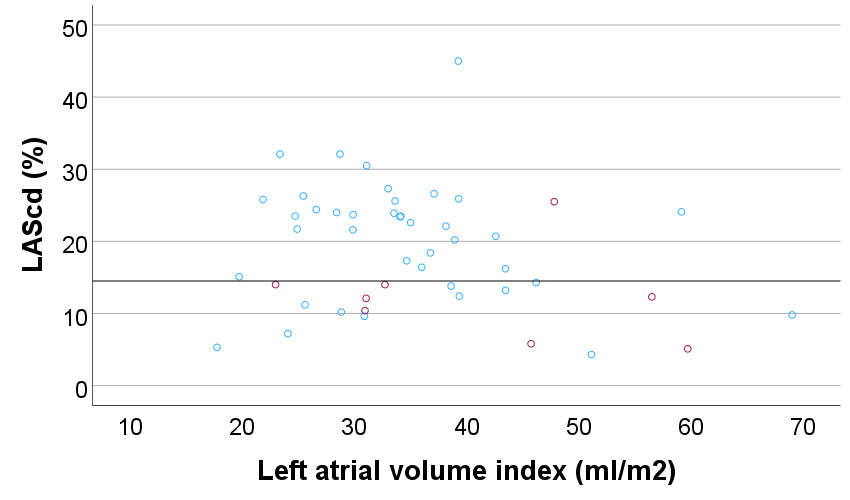
**
